# “I love having a healthy lifestyle" – a qualitative study investigating body mass index trajectories from childhood to mid-adulthood

**DOI:** 10.1186/s40608-019-0239-3

**Published:** 2019-05-06

**Authors:** M. J. Sharman, K. A. Jose, A. J. Venn, S. Banks, J. Ayton, V. J. Cleland

**Affiliations:** 1 Menzies Institute for Medical Research, University of Tasmania, Hobart, Tasmania Australia; 2 School of Social Sciences, University of Tasmania, Hobart, Tasmania Australia; 3 School of Medicine, University of Tasmania, Hobart, Tasmania Australia

**Keywords:** Diet, food and nutrition, Body weights and measures, Body weight, Over nutrition, Exercise, Health promotion

## Abstract

**Background:**

Children with overweight or obesity are at greatly increased risk of experiencing obesity in adulthood but for reasons generally unknown some attain a healthier adult weight. This qualitative study investigated individual, social and environmental factors that might explain diverging body mass index (BMI) trajectories. This knowledge could underpin interventions to promote healthy weight.

**Methods:**

This 2016 study included participants from three adult follow-ups of children who (when 7–15 years) participated in the 1985 Australian Schools Health and Fitness Survey and provided BMI data at each time point. Trajectory-based group modelling identified five BMI trajectories: stable below average, stable average, increasing from average, increasing from very high and decreasing from very high. Between six and 12 participants (38–46 years) from each BMI trajectory group were interviewed (*n* = 50; 60% women). Thematic analysis guided by a social-ecological framework explored individual, social and environmental influences on diet and physical activity within the work setting.

**Results:**

A distinct approach to healthy behaviour was principally identified in the stable and decreasing BMI groups – we term this approach “health identity” (exemplified by “I love having a healthy lifestyle”). This concept was predominant in the stable or decreasing BMI groups when participants explained why work colleagues seemingly did not influence their health behaviour. Participants in the stable and decreasing BMI groups also more commonly reported, bringing home-prepared lunches to work, working or being educated in a health-related field, having a physically active job or situating physical activity within and around work – the latter three factors were common among those who appeared to have a more distinct “health identity”. Alcohol, workplace food culture (e.g. morning teas), and work-related stress appeared to influence weight-related behaviours, but generally these factors were similarly discussed across all trajectory groups.

**Conclusion:**

Work-related factors may influence weight or weight-related behaviours, irrespective of BMI trajectory, but the concept of an individual’s “health identity” may help to explain divergent BMI trajectories. “Health identity” and its influence on health behaviour warrants further exploratory work.

**Electronic supplementary material:**

The online version of this article (10.1186/s40608-019-0239-3) contains supplementary material, which is available to authorized users.

## Background

In 2013, nearly one third of adults and children worldwide (~ 2 billion) were estimated to be living with overweight or obesity [1]. This is of public health significance because weight and comorbidity are positively correlated, thus resulting in substantial personal and societal cost [2]. In 2012, obesity was ranked as the third most expensive human generated social burden, just behind smoking and armed combat [3]. In Australia, 63.4% of adults (≥ 18 years) and 25.8% of children (2–17 years) were estimated to be affected by overweight or obesity in the period 2014–15 [4, 5]. Recent high quality data are lacking, but in 2005 the direct cost of excess weight to the Australian economy was estimated at $21 billion (AUD) [6].

Initiatives to prevent or reduce unhealthy weight gain are critical for public health, but no country as yet has been able to redress its obesity epidemic despite efforts to do so (e.g. through taxes on non-essential energy dense foods [7], improved food labelling [8], advertising restrictions [9]). At an individual level, weight loss through lifestyle modification or medical management is generally modest at best and difficult to sustain, especially in individuals experiencing higher than normal weight [10–13]. The complex causal contribution of individual, social and environmental factors to overweight and obesity likely explains its commonly intractable nature and highlights the importance of avoiding unhealthy weight gain trajectories [14].

Taking a life-course perspective, childhood antecedents of adult overweight/obesity include individual (e.g. birth weight) and social (e.g. parental obesity, low socio-economic status) factors [15, 16]. Further, children with overweight or obesity are at much greater risk of having overweight or obesity as adults compared with children of healthy weight. For example, in one prospective cohort study, the relative risk of having obesity in adulthood was 4.7 times greater in boys with obesity and 9.2 times greater in girls with obesity compared with children of normal weight [17]. Despite the much greater risk of obesity, some children with excess weight achieve a healthy weight in adulthood [17]. Transitioning from unhealthy to healthier weight trajectories is associated with lower health care costs and more favourable cardio-metabolic health profiles [6, 18–20]. For example, children with overweight or obesity who do not have obesity by adulthood have similar cardio-metabolic profiles to those classified as healthy weight in childhood who avoid obesity in adulthood [20]. Whilst the health risks associated with differing weight trajectories and the childhood predictors of adult overweight or obesity are generally known, there are important knowledge gaps regarding why some people remain on lower risk weight trajectories across the life-course, or divert from high to lower risk.

The social-ecological framework is useful for improving understanding of health-related behaviours, specifically dietary and physical activity behaviours related to weight, ideally leading to more appropriate intervention [21, 22]. These models posit that individual (e.g. psychological/cognitive factors), social (e.g. family, peers, work colleagues) and environmental (e.g. access to healthy foods and physical activity opportunities) factors interact to influence behaviour [21, 22]. While some studies have used these models to investigate predictors of weight status, no research has employed a social-ecological framework to identify drivers of diverging weight trajectories from childhood to adulthood [23–25]. Few studies have attempted to understand the underlying reasons for diverging weight trajectories from childhood into adulthood.

Most adult Australians aged 35–54 years’ work over 30 h per week, making work a key influence in daily life [26]. Workplaces have social and environmental dimensions likely to elicit common and diverging responses among individuals. For instance, smoke-free workplaces positively impact the smoking behaviour of some but not all employees and some professions appear to be associated with poorer health behaviours than others [27, 28]. From a broad pragmatist perspective, which seeks to investigate the realities of ordinary life to create knowledge for practical use, this raises an important question [29]. What drives different health-related behaviours among individuals as exposed through the work setting? Thus, the objective of this qualitative study was to use a social-ecological model to investigate individual, social and environmental factors within the work setting that may influence diverging weight trajectories from childhood to mid-adulthood. Filling this knowledge gap could lead to more effective weight-related interventions.

## Methods

### Design and sampling

**Fig. 1:**
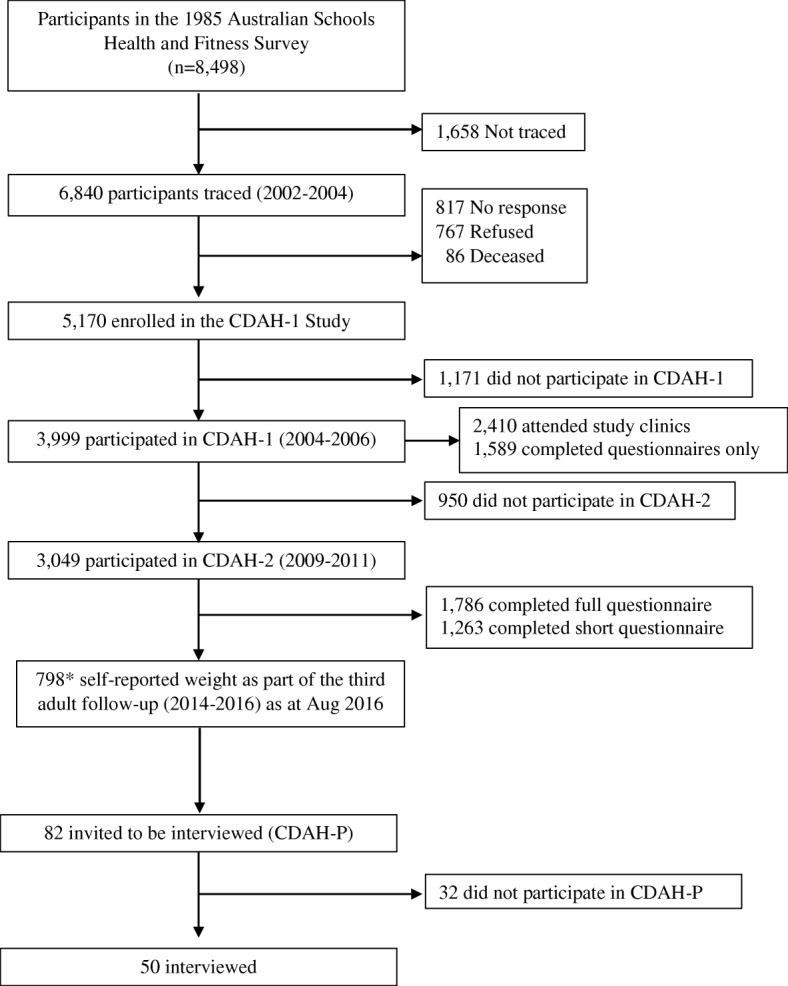
Participant flowchart. *NB: This is not the total sample of participants in the third adult CDAH follow-up, as data collection were ongoing at the time of sampling and recruitment

This qualitative study guided by pragmatism, used purposive sampling to select participants to undertake semi-structured phone interviews. Participants were characterised as being on different body mass index (BMI) trajectories from childhood to mid-adulthood [29]. These participants were drawn from the Childhood Determinants of Adult Health (CDAH) study a prospective quantitative study which has involved three adult follow-ups of a nationally representative cohort who (when aged 7–15 years) completed the 1985 Australian Schools Health and Fitness Survey (*n* = 8498) (Fig. 1) [30–32]. BMI trajectory-based group modelling used measured and/or self-reported height and weight data from four time points (1985, 2004–6, 2009–11, 2014–16) [33] to identify distinct BMI trajectories. Where measured height and weight data were unavailable, a correction factor was applied using a linear regression model of self-reported data, collected from participants who had both clinical and self-reported measures available [17]. Five BMI trajectories were identified among the CDAH cohort: stable below average, stable average, increasing from average, increasing from very high, and decreasing from very high.

### Recruitment

Twenty people (women and men) were randomly selected from each of the five CDAH BMI trajectory groups (except for one group, increasing from very high, which only had 19 participants and thus all were selected) to form the sampling frame. Letters of invitation were mailed every three to 4 weeks in batches of 25 (five from each group), until the initial target of 50 participants had been confirmed through telephone follow-up. The intention was to have approximately ten participants per BMI trajectory group, although a minimum of six participants was considered adequate given the study aim and analytic approach [34]. Verbal consent was audio-recorded preceding the interview questions. By the 40th interview there was an absence of new themes emerging. An additional ten interviews were conducted to ensure that data saturation had been reached overall and that there was reasonable representation across the BMI trajectory groups. Subsequently, no more than 50 interviews were considered necessary [35].

### Measures

Demographic information for each interview participant was attained from the 2014–2016 CDAH follow-up survey, supplemented by information obtained from the interviews. The interview schedule (Additional file 1) was underpinned by the social-ecological theory of health behaviours, where individual (e.g. values, beliefs), social (e.g. work colleagues, family, peers) and environmental (e.g. work, school, neighbourhood) influences on weight-related behaviours (e.g. diet and physical activity) across the life-course were explored in telephone interviews [21, 36]. Pilot testing the interview schedule on one man and one woman external to, but of the same age group as the cohort, resulted in minor changes only. To ensure consistent data collection, one experienced author (SB), blinded to trajectory group, conducted all interviews, which were audio-recorded. In this manuscript, we present the findings relevant to the work setting only.

### Data analysis

The interviews were transcribed verbatim and thematically analysed, using a constant comparative approach (a grounded theory method) facilitated by use of NVivo 11 (QSR International, Doncaster, Victoria, Australia) [37]. A codebook, developed during data collection informed by the social-ecological theory of health behaviours, guided a team coding approach involving authors KJ, SB, VC, JA and MS [21, 36, 38]. Coding consistency was checked on two occasions using a common interview, resulting in some refinement to the codebook. Regular meetings occurred during the six-month team coding phase (November 2016–April 2017) to facilitate data coding and to discuss emerging themes. On completion of the preliminary analysis all coding was reviewed by MS for thoroughness and relevance. For the purpose of the thematic analysis, the stable below average and stable average groups were collapsed into one group (referred to as “stable”), which was compared with the remaining three trajectory groups (referred to in the presentation of the thematic analysis as average increasing, high increasing, high decreasing). Meetings with all authors continued until there was consensus that the data had been comprehensively analysed, and no new themes were emerging. An audit trail was kept throughout the analysis phase and included the interview schedule, transcripts, codebook, notes on research team meetings, memos and reflective notes. Transcribed interviews were not returned to participants for checking.

## Results

Fifty participants were interviewed after approaching 83 prospective participants (response rate 60%; lowest response rate seen in the increasing from very high group at 46%). Lack of response to telephone or email messages was the main reason for non-participation (*n* = 18). Interviews (average duration 51 min, range 25–94 min) were conducted from August to November 2016. No participants withdrew from the study.

Participants’ characteristics collected from the 2014–16 CDAH follow up and interviews are described in Table 1. In summary, participants (21 men; 29 women) were middle aged adults, of which most were employed (92%) with dependents (76%), and many had a university qualification (56%).

**Table 1:** Participants characteristics by BMI trajectory groups

*Characteristic*	*All (N = 50)*	*Stable below average (n = 10)*	*Stable average (n = 12)*	*Increasing from average (n = 11)*	*Increasing from very high (n = 6)*	*Decreasing from very high (n = 11)*
Age (years)						
Mean (range)	42 (38–47)	43 (39–45)	43 (39–46)	42 (38–46)	41 (39–45)	42 (38–47)
Sex						
Female n (%)	29 (58)	5 (50)	7 (58)	7 (64)	5 (83)	5 (45)
BMI (kg/m^2^) at four time-points						
^1^ Mean (SD)	19.8 (3.4)	17.1 (1.2)	20.4 (2.5)	18.0 (3.4)	20.8 (3.1)	23.0 (3.1)
^2^Mean (SD)	28.4 (6.1)	21.7 (2.0)	26.6 (1.8)	32.8 (3.3)	39.3 (4.0)	27.0 (3.6)
^3^Mean (SD)	29.3 (6.8)	22.2 (3.1)	27.4 (2.5)	33.2 (3.6)	42.5 (3.4)	28.0 (3.4)
^4^Mean (SD)	29.7 (7.2)	22.0 (1.8)	27.3 (2.8)	37.0 (4.4)	40.9 (4.0)	27.2 (3.4)
Highest level of education^a^						
High n (%)	28 (56)	7 (70)	5 (42)	5 (45)	1 (17)	10 (91)
Medium n (%)	8 (16)	1 (10)	1 (8)	4 (36)	2 (33)	0 (0)
Low n (%)	14 (28)	2 (20)	6 (50)	2 (18)	3 (50)	1 (9)
Employed ^b^						
n (%)	46 (92)	9 (90)	11 (92)	10 (91)	5 (83)	11 (100)
With dependents						
n (%)	38 (76)	7 (70)	9 (75)	9 (82)	3 (50)	10 (91)

^1^Based on measured height and weight in 1985 ^2^ Based on self-reported and measured height and weight in 2004–6 follow up^3^ Based on self-reported weight in 2009–11 follow up ^4^ Based on self-reported weight in 2014–16 follow up

^a^Low: school only; Medium: Certificate/diploma, trade/apprenticeship; High: Bachelor/Higher degree

^b^For two participants work status was uncertain, but both were counted as unemployed because unemployment was implied in the transcripts

Key: BMI body mass index

For this manuscript, the social-ecological framework of analysis was situated within the work setting, where individual, social and environmental factors were compared across trajectory groups. Most participants appeared to be working full-time and working in administrative/office-based roles. Two participants were not working for health reasons and the transcripts implied they were receiving disability support pensions – data from these participants were included in the thematic analysis as appropriate (e.g. reference to work history). Seven key themes emerged from the thematic analysis, exposing individual, social and environmental influences of weight-related behaviours and differences between BMI trajectory groups. The major finding was that an individual’s “health identity” may play a role in shaping BMI trajectories. The main themes are discussed below and summarised in Table 2. Quotes cited are from participants, with gender and membership of BMI trajectory group noted.

**Table 2:** Summary of key themes emerging from the social-ecological framework of enquiry within the work setting, with references to health identity highlighted

*Social-ecological level*	*Work-related factor*	*Summary finding*
Individual	Physical activity	Enduring or predominant active transport use, or a history of active transport use was more commonly referenced in the stable and decreasing groups. Participants in the stable and decreasing groups talked about prioritising physical activity around work obligations more commonly than participants in the other BMI trajectory groups. Among participants whose distinct “health identity” appeared to be influencing their health behaviours, reference to physical activity within and around work was common.
	Stress	Except from within the high increasing BMI trajectory group, there were several references made to work stress negatively affecting diet or the amount of physical activity conducted.
	Alcohol consumption	There were generally no differences across BMI trajectory groups regarding the discussion of work and alcohol, although reference to binge or regular drinking was more common among participants categorised as stable or decreasing.
Social	Colleagues	Generally appeared to have little influence across all BMI trajectory groups. Reference to a distinct “health identity” as a reason why work colleagues were not influential was common in the stable and decreasing groups but not the other groups.
Environmental	Workplace health promotion	Generally appeared to have little influence across all BMI trajectory groups. “Health identity” referred to in the stable group as a reason for lack of influence of workplace health promotion.
	Field and type of work	Working in or being educated in a health-related field as a positive influence on health behaviours was referred to in the stable and decreasing groups only. All participants who discussed this relationship also suggested that their positive health behaviours were influenced by their “health identity”. Participants in the stable group more commonly referred to current and historical work roles that included physical activity.
	Food culture	Across all BMI trajectory groups, there was common reference to current or previous challenges associated with regular morning teas and the presence of vending machines or freely accessible sugary snacks. No systematic differences were found between BMI trajectory groups for the type of foods bought for lunch. However, those in the stable and decreasing groups more commonly indicated that they brought a prepared lunch from home to work.

### Work colleague influence

Across all BMI trajectory groups, the perceived impact of work colleagues on dietary or physical activity behaviours was limited. Work colleagues appeared to be “not at all” (female, high increasing) influential or only “somewhat influential” (male, stable). Where references were made to work colleagues being influential on health behaviour in a positive way, sometimes the statements made were unconvincing or the influence appeared transient, “So, through osmosis I’m just letting that attitude [eating healthily and exercising] come to me” (male, average increasing). While this seeming lack of work colleague influence was evident across all BMI trajectory groups, the reason for the lack of influence differed. For over a third of participants in each of the stable and decreasing groups, less susceptibility to work colleague influence could be attributed to a distinct “health identity”:

I don’t really take that much influence from my work colleagues or friends particularly, it's just something I've grown up knowing it's sensible to have a healthy lifestyle and a good diet and exercise is something you need to stay physically well. (male, stable)

In contrast, this type of explanation was only apparent for one person in each of the increasing groups.

### Workplace health promotion strategies

Over a third of all participants said that their workplace supported or initiated health related promotion strategies (e.g. Weight Watchers at work, company marathons, 10,000 steps challenges) and provided supportive facilities (e.g. showers, gyms) or equipment (e.g. FitBits, sit-stand desks). No systematic differences were discernible between the BMI trajectory groups, with the collective discussion suggesting that this aspect of the workplace was not particularly influential on health behaviours. One participant, who said that health promotion initiatives at their workplace were influential, also indicated that ultimately, the responsibility was their own, “They [the workplace] have been influential, but then I’ve got to be in the right mindset to take advantage of that” (female, average increasing). One participant from the stable group explained that the generous health-related support available at her workplace was not very influential because, “I probably do that outside of work” (female). This kind of explanation may again signal that a distinct “health identity” could be important in driving positive health behaviours.

### Field and type of work

A theme that was only discussed in the stable and decreasing groups (by five participants) was that working in or having been educated in a health-related field had a positive impact on health behaviours, although there was variation in the apparent magnitude of effect, “I do work in a pathology service, so I guess health is on our mind in a way” (female, high decreasing); and “You’re just educated [nursing] you know that you can’t eat junk food all the time and maintain a healthy biochemistry and health lifestyles. So just don’t do it” (female, high decreasing). All five of these participants working in or educated in a health-related field also insinuated that their positive health behaviours were influenced by their distinct “health identity”.

Participants categorised as having a stable BMI trajectory more commonly referred to current and historical occupational physical activity compared with the other groups, “But I do a lot of walking, because I’m moving things around, shifting things from one side to the other, I stand up a lot. It’s not a very stationary job” (female) and “I’ve always been pretty outdoorsy and I’ve always chosen employment that’s been predominantly outdoors [e.g. landscaping]” (male). Although a few participants from the normal increasing and high decreasing groups also referred to working roles that included physical activity, this theme was more common among those from the stable BMI trajectory group.

Some participants discussed the impact of job type on their health which was highlighted when moving to or away from an active role, “… so I can definitely tell now that I’ve had to go back out to workshop [to cover a staff shortage] that my fitness level is nowhere near what it used to be” (male, stable) and “I found that, where now I’m back in physical work…I’ve noticed now I’ve trimmed down quite a bit” (male, average increasing).

### Situating physical activity within and around work

The most commonly reported mode of commuting to work was by motor vehicle for participants across all BMI trajectory groups. Enduring or predominant active transport use, or a history of active transport use was more commonly referenced in the stable and decreasing groups, “I’ve been riding my bike [to and from work] now for only about eight years, I think, so I started that pretty late but I’ve stuck with it” (female, stable). Active commuting (e.g. by bike) was referred to by only one participant in the increasing groups.

With the exception of participants categorised as high increasing, a few participants talked about taking deliberate measures to mitigate the sedentary nature of their jobs, “Usually I try and go to the gym at lunchtime at least, depending on how busy I am, two to four times a week during lunchtime so at least I’m up and moving” (female, average increasing).

Participants in the stable and decreasing groups talked about prioritising physical activity around work obligations more commonly than participants in the other BMI trajectory groups, “I’ve tried to make sure that it [work] doesn’t influence my set physical activity, like I’m still making sure I get my netball game in every week” (female, stable). Prioritising physical activity seemed to remain important despite seemingly busy lives, even if physical activity was viewed as a chore, “It’s [physical activity] a forced thing, though” (female, stable).

Among participants whose distinct “health identity” appeared to be influencing their health behaviours, reference to physical activity was a common element. All of these participants made statements about: situating physical activity within or around their work commitments, “Now I have to really take that time and get up at six in the morning and walk” (female, stable); or actively commuting to work; or having an active job (e.g. nursery work) or a job that included at least some physical activity, “I work in a lab, so it’s very rare that you’d be sitting at your desk all day” (male, high decreasing).

### Workplace food culture

Across all BMI trajectory groups, there was common reference to current or previous challenges associated with regular morning teas, and the presence of vending machines or freely accessible sugary snacks (confectionary jars and chocolates), “They’re the tough times; the morning teas and the congratulations….” (female, high increasing) and “You feel like some of that junky food is far more available than what I have seen in other [workplaces]. That’s a bit more of a challenge for me I find” (female, stable). Two participants (one each in the stable and high decreasing groups) believed access to chocolates in the workplace had contributed to weight gain, while another said that chocolates being readily available at their workplace “drives me nuts” (female, high increasing). One participant referred to the influence of a soft drink vending machine at work, “But maybe a year or two ago I might have had one or two cokes a week at work, just for a little while, because we had a coke machine there” (male, stable). Restricting morning teas to once per month was considered helpful by one participant because, “… you don’t get sucked into eating rubbish every day…” (male, high decreasing). A few participants in the average increasing and high decreasing groups indicated that having a free healthy snack available at work was a positive workplace initiative. Another said that the workplace had little influence on eating behaviours, “I think you ended up big because you have a liking for food. So it doesn’t matter where you work you’re still going to eat” (female, high increasing).

No systematic differences were found between BMI trajectory groups for the type of foods bought for lunch. However, in contrast to participants in the increasing BMI trajectory groups, those in the stable and decreasing groups more commonly indicated that they brought a prepared lunch from home to work.

### Work-related stress

A fifth of participants across the stable, average increasing and high decreasing groups attested to the impact work-related stress had on their health or health behaviours, “So, that definitely has an impact on your health, the amount of hours you’re doing and the stress of the job” (male, stable). Three participants identified work stress as contributing to weight gain, with weight loss in response to work stress reported by one participant (from the stable BMI trajectory group). Except from within the high increasing BMI trajectory group, there were several references made to work stress negatively affecting diet or the amount of physical activity conducted. For example:

…when I got onto a big and stressful project at work, that was when my level of exercise decreased and similarly the quality of food I was eating was decreasing as well. And then I’d always seemed to put on weight when I got very stressed at work... (male, high decreasing)

### Alcohol consumption and work

The discussion across the BMI trajectory groups indicated that work acted as both a barrier and enabler to alcohol consumption. Work commitments appeared to moderate alcohol consumption during the week, followed by greater consumption on the weekends:

I don’t touch anything through the week but on Friday I might have a couple of beers. I might have a few on Saturday with some mates... Sunday… I might have a couple of beers and I never have too much because of work on Monday. (male, average increasing)

A couple of participants associated more alcohol consumption with increased earnings. Reference to binge or regular drinking was more common among participants categorised as stable or decreasing and was occasionally referred to as a strategy for managing work-related stress, “You get to the end of the week, you’ve been stressed out all week, it’s a Friday and you have a drink to relieve – it’s like blowing off steam…” (male, stable).

## Discussion

Using work as a setting for exploration, this novel study has exposed a concept we refer to as “health identity” (a distinct approach to healthy behaviours) that may influence diverging BMI trajectories from childhood to mid-adulthood. For instance, those who had a stable or decreasing from high BMI trajectory more commonly reported a commitment to healthy behaviours (such as a balanced diet and regular physical activity) as an explanation for certain work-related factors (e.g. colleagues) seemingly having little influence on their lifestyle choices. The strength of an individual’s “health identity” may be an important and unrecognised element contributing to varying vulnerability to the obesogenic environment. It may also explain why the impact of health promotion programs in the work setting has been mixed [39]. An individual’s identity is influenced by personal and social contexts, including gender, culture, leisure and work and identity can influence mental or physical health [40–43]. It is increasingly recognised that leisure choices and lifestyles in contemporary society are linked with identity formation [44]. The concept of a health identity is congruent with research findings that greater physical activity is associated with a stronger “physical identity”. For instance, children and adolescents’ self-concepts can explain how much effort and time they devote to various physical activities [45]. As social and leisure practices such as eating and physical recreation have become progressively associated with health, it is plausible that some individuals will develop a distinct “health identity” that is reflected in weight-related behaviours [46]. The relationship between “health identity” and health outcomes, specifically obesity redress and prevention, is worthy of further investigation.

Other health behaviours and outcomes such as smoking, alcohol consumption, depression, obesity and eating behaviour appear to spread in social networks [47–51]. It is therefore plausible that the strength of an individual’s “health identity” may also be influenced by social networks. Corroborating our findings, other studies have found that co-workers appear to have little influence on health outcomes or behaviours [48, 50, 51]. The spread of health behaviours across social networks seems most common through family and friends, suggesting that further exploratory work investigating the relationship between “health identity” and these social groups would be worthwhile [47–51]. Given the wide-reaching benefit of preventing unhealthy lifestyle behaviours, if establishing a strong “health identity” is a key driver of healthier BMI trajectories across the life-course, then interventions focused on the childhood years (e.g. through positive parenting and school- based approaches) will be necessary.

Social-ecological models posit that a range of individual, social and environmental factors interact to influence health behaviours [21, 22]. Within the work setting, we also found that, individual-level factors more so discriminated divergent BMI trajectory groups, than social and environmental level factors, particularly in respect to physical activity and its relationship to the work setting. This finding is consistent with other studies exploring the relative influence of broader individual, social and environmental factors on physical activity, where behaviours appear largely driven by individual-level factors such as self-efficacy and enjoyment of physical activity [52–55].

Situating physical activity in and around work (e.g. active commuting, physically active work types, using breaks to engage in physical activity, physical activity immediately before/after work) was a predominant theme in those classified with a stable BMI trajectory and was common among all who presented with a distinct “health identity” (both from within the stable and high decreasing BMI trajectory groups). This may indicate that these individuals have identified work as an ‘anchor’ upon which to leverage their physical activity patterns and given the typically regular nature of work, it may have played an important role in habit formation. Physical activity is important for the prevention and treatment of weight gain and obesity [56], so determining the drivers of physical activity as a long lasting habit is an important knowledge gap to fill. Strategies that promote situating physical activity around work (e.g. active commuting, physical activity at lunchtime), rather than within work, may also be warranted.

This study also exposed several public health concerns that were relevant to participants in all BMI trajectory groups, such as matters raised about the impact of a negative workplace food culture. Removing vending machines, reducing the regularity of morning teas or providing only healthy morning tea options and removing free sugary snacks may be helpful, easily implementable and inexpensive initiatives to mitigate obesogenic workplace environments. Other important health-related issues raised across all BMI trajectory groups, concerned the perceived association between work stress and poorer health behaviours (e.g. eating more convenience food when stressed at work) or outcomes (e.g. increased weight) and the relationship between work and risky alcohol behaviour (e.g. binge drinking at the end of the working week to relieve stress).

### Limitations

Although data saturation was determined by the 50th interview, it is possible that other relevant themes were not discussed. Social desirability pressures may have influenced participant responses, but this possibility may have been reduced by use of telephone interviewing which can enable a greater sense of anonymity and less social pressure compared with face to face interviewing [57]. Recall error may have also influenced the results given that participants were middle-aged adults and were asked to recollect details across their full work history. It is also possible that a person’s BMI trajectory may have affected their perceptions on the influence on work-related factors. Meaningful comparisons could not be made between individuals by work hours because of incomplete data — while work emerged as an important setting for investigation, it was not the central focus of the study, so these data were not collected systematically. The relationship between longer work hours, work stress and lifestyle behaviours requires further systematic investigation. Another possible limitation is that the BMI trajectory of individuals may have been misclassified, because of the use of some self-reported weight and height data, because of weight fluctuations in the intervening years between data collection not truly reflecting ‘usual’ weight, or because of the method chosen (group-based modelling) to generate trajectories. Potential for misclassification was minimised due to the statistical correction factors applied to the self-report data. Further, based on the descriptive data, the BMI trajectory groups have face validity (i.e. mean BMI at the final time point was lowest in the stable and decreasing groups and highest in the increasing groups). Finally, the generalisability of the findings is unknown, but the study has yielded important new insights from a sample of men and women of diverse BMI trajectories and socioeconomic status.

### Strengths

The sample for this novel study was drawn from a large and high-quality prospective cohort study containing mostly measured height and weight data from four discrete time points over a 30-year period from childhood to mid-adulthood. This enabled the classification of participants into five discrete BMI trajectories. Few such studies exist, providing a unique opportunity to use quantitative data for the purposive selection of participants in this study. The use of a single blinded interviewer and the rigorous team coding approach used for data analysis add to the strengths of this research. Finally, this study was underpinned by a theoretically driven approach within a social-ecological framework, which is important for establishing a comprehensive understanding of the factors influencing weight-related behaviours.

## Conclusion

Work-related factors may influence weight or weight-related behaviours, irrespective of BMI trajectory. An individual’s “health identity” may be an important unrecognised element influencing diverging BMI trajectories across the life-course. The concept of “health identity” warrants further exploratory work as it may play a role in establishing more favourable, or remediating unfavourable, BMI pathways.

## Additional file

Additional file 1: Title of data: Interview schedule. Description of data: List of interview questions. (DOCX 21 kb)

## Abbreviations

BMI: Body mass index
CDAH: Childhood Determinants of Adult Health
