# Supplementary material for: “I love having a healthy lifestyle" – a qualitative study investigating body mass index trajectories from childhood to mid-adulthood
Source: BMC Obes. 2019 May 6;6:16. doi: 10.1186/s40608-019-0239-3 (PMC6501298; doi:10.1186/s40608-019-0239-3)
Supplement: Supplementary file 1 — Title of data: Interview schedule. Description of data: List of interview questions. (DOCX 21 kb) [file 40608_2019_239_MOESM1_ESM.docx]

**CDAH Pathways Study – Semi-Structured Interview Schedule**

We are interested in hearing today about your lifestyle – in particular the things you eat and drink, your physical activity, and weight – over the years. We are interested in your journey, also known as a trajectory, from childhood, through adolescence and into adulthood, and in any key events in your life that may have impacted on the things you eat and drink, your physical activity and your weight. When I talk about physical activity, I am referring to this quite broadly – so things you do around the home like gardening and vacuuming, things you do at work like lifting or walking, ways of getting from one place to another like walking or cycling, and things you do in your leisure, like walking or playing sport. Other lifestyle behaviours such as smoking or alcohol may also be important to you and I am happy for you to tell me about these if you feel they are relevant, but we are mostly interested in your diet, physical activity and weight. Please remember that you are the expert here, there are no right or wrong answers, and I am interested in hearing your unique story. If there are any questions you prefer not to answer, there is no obligation to do so.

1. Could you tell me what leading a healthy lifestyle means for you? Has this changed over time? How important is leading a healthy lifestyle to you?
2. Thinking mostly about what you eat and drink, and your physical activity, can you tell me about your lifestyle since childhood *(cover childhood, adolescence, early adulthood, and now)?* Is your lifestyle more or less healthy now than in the past? We are also interested in hearing about your weight since childhood, can you tell me a bit about that?
3. Sometimes there are significant events or changes in our lives that impact on lifestyle. Can you think of one event or change that has impacted on your lifestyle behaviours, particularly in relation to what you eat and drink, and your physical activity?

*(this might be leaving school, employment, marriage/separation, children, health/disability) (please tell us about any things that changed your lifestyle and how it changed) (if there are lots of things, can you tell us in detail about one?)*

1. Next I would like to talk about family.^[[1]](#footnote-1)^
   1. First, let’s talk about the family you grew up in – your parents, siblings and any extended family members that played an important role in your life. Can you tell me a bit about your family, and their lifestyle? What value did your family place on leading a healthy lifestyle? What influence has this had on your lifestyle, now and in the past? *(please also tell us about whether this has changed over the years)*
   2. (if relevant): Let’s now talk about your own family now – particularly your partner/spouse but also if relevant your children. Can you tell me a bit about your family, and their lifestyle? What value does your family place on leading a healthy lifestyle? How does this relate to your own lifestyle, now and in the past? *(and has this changed over the years?)*
2. Next I would like to talk about your friends and, if relevant, work colleagues. Can you tell me a bit about your friends and work colleagues’ lifestyles with respect to health? What value do your friends/colleagues place on leading a healthy lifestyle? What influence has this had on your lifestyle, now and in the past? *(and please tell us whether this has changed over the years, and how).*
3. Thinking about the places you have lived and worked over the years, how do you think this has influenced your capacity to lead a healthy lifestyle? *(some influences might be the availability/accessibility of healthy food, availability/accessibility of places to be active) (can you tell us if this has changed over the years?)*
4. What has been or is the biggest challenge for you with respect to eating well and being active (and managing weight if relevant)?
5. Moving forward, tell me about your plans or intentions over the next few years in relation to your lifestyle.
6. Is there anything else that we haven’t covered today that you would like to tell me, in relation to your life, lifestyle or weight?

1. For questions 4-6, please provide a specific example that illustrates the point. For example: can you talk about what a typical family meal might have been like as a child. How does this compare to a typical evening meal now? Can you talk about the types of things your family would do for leisure when you were a child? How does this compare to the types of leisure pursuits you engage in now? [↑](#footnote-ref-1)
